# Differential Expression of Serum Exosomal miRNAs in Breast Cancer Patients and Healthy Controls

**DOI:** 10.34172/apb.2022.088

**Published:** 2021-10-06

**Authors:** Rahim Asgari, Jafar Rezaie

**Affiliations:** Solid Tumor Research Center, Cellular and Molecular Medicine Institute, Urmia University of Medical Sciences, Urmia, Iran.

**Keywords:** Breast cancer, Exosomes, miRs, Biomarker

## Abstract

***Purpose:*** Breast cancer has become as a serious public health concern worldwide. Breast cancer cells release exosomes into the circulatory system, which are easily accessible for further analysis like cancer diagnosis. In this study, we aimed to investigate expression of circulating exosomal miRNAs (miRs) in the serum of individuals with breast cancer and healthy controls.

***Methods:*** Exosomes were collected from serum samples using a commercial kit and characterized by scanning electron microscopy (SEM) and flow cytometry analysis. Expression of miRs such as miR-21, miR-155, miR-182, miR-373, and miR-126 were evaluated by real-time PCR.

***Results:*** The result showed that the expression level of exosomal miR-21, miR-155, miR-182, and miR-373 in the serum of breast cancer patients was higher than of those controls (*P*<0.05). However, expression of miR-126 did not change between breast cancer and control individuals (*P* > 0.05).

***Conclusion:*** Our results showed a different miRs expression pattern between breast cancer and healthy samples, supposing potential biomarkers for breast cancer. Further studies focusing on these miRs are required to confirm our findings.

## Introduction

Breast cancer is prevalent with the highest incidence globally, causing mortality among women.^1^ Despite significant progress in early detection, diagnosis, and therapy, this cancer is the main causes of cancer-related mortality among women due to relapse metastatic illness.^2^ As a result, early detection of breast cancer is particularly vital for improving the clinical outcomes in patients with breast cancer.^3^ Thus, despite the development of the imaging methods (i.e., mammography and echography) and diagnostic biomarkers, they are not yet perfectly suitable, mainly due to such challenges as being time-consuming and costly, and limitation in identifying distal disease.^4,5^ In the case of cancer, liquid biopsy is increasingly becoming a vital factor for early detection, diagnosis, recurrence, and even therapeutic management.^6^ Liquid biopsy, an encouraging alternative to traditional tissue biopsies, represents a novel approach in precision oncology to overcome present challenges associated with solid biopsies. In addition, liquid biopsy has been thought of as a key factor in understanding the possible mechanisms behind metastatic processes happening in blood.^7^ Therefore, liquid biopsies could be a useful tool for guiding cancer treatment methods and possibly even screening for tumors, which are not detectable on imaging method.^8^ Exosomes are 30-150 nm vesicles releasing from cells that present in almost body fluids including blood.^9^ Previous studies showed that tumor cells produce exosomes that can reach recipient cells and tissues, and therefore promote tumorigenesis.^9,10^ Exosomes contain different types of biomolecules including miRNAs (miRs), mRNAs, DNA fragments, proteins, lipids, and signaling mediators promoting tumor growth, metastasis, and angiogenesis.^10^ Recently, researchers have shown an increased interest in application of exosomal miRs in cancer. As exosomes are bio-envelope, exosomal miRs are sheltered from nucleases and shear stress, and these properties make these molecules potential biomarkers for cancer detection. Studies of miRs show that distinct miRs unusually expressed in tissues, blood, and cancer cells, which proposed as new breast cancer biomarkers.^11,12^ However, these results have not yet been confirmed to be successful, mostly due to confusing factors affecting levels of exosomal miRs and doubting their potential as cancer biomarkers.^13^ At present work, we studied the expression pattern of miRs isolated from exosomes of serum samples of patients with primary breast cancer.

## Materials and Methods

###  Materials

 ExoQuick exosome isolation kit was purchased from SBI (USA). Primary anti-CD63 antibody was obtained from Santa Cruz Company (USA). Secondary anti- mouse IG-FITC was provided from BioLegend Company (USA). MicroRNA assays and cDNA synthesis kits were purchased from Stem Cell Technology Research Center (Tehran, Iran).

###  Plasma isolation

Sera were collected from newly identified breast cancer patients (n = 7) and co-aged normal individuals (n = 7). Individual’s characteristics at the time of first diagnosis of breast cancer presented in Table 1. All donors have filled an informed consent form. Blood samples were collected and then centrifuged at 1200 g for 10 minutes. Sera extracted from the supernatant and then moved into another tube. After centrifuging at 1800 g for 15 minutes, sera were stored at −80°C for downstream experiments.

**Table 1:** Characteristics of breast cancer patient and control individuals

	**Age**	**Her2**	**Estrogen receptor**	**Progesterone receptor**	**Grade**
Breast cancer	51.3 ± 7.85	+	+	+	I,II,III
Control	48.25 ± 5.85	-	-	-	-

###  Exosomes isolation

 Serum exosomes were purified using ExoQuick exosome isolation kit according to the manufacturer’s recommendation. In brief, 1 mL each serum sample was mixed with 240 µL ExoQuick solution and kept for 30 min at 4°C. Next, samples were centrifuged at 1500 × g at 4°C for 30 minutes. Exosomes pelleted at the bottom of the tube and then resuspended in 200 µL PBS for exosomes characterization or in 350 µL Lysis Buffer for miRs extraction. After adding Lysis Buffer, samples were kept for 5 min and then 200 µL ethanol was added, after that the mixture was transferred into RNA column and centrifuged at 13 000 rpm for 1 minutes. After washing, samples were dried at 13 000 rpm for 2 minutes. In keeping, 30 µL elution reagent was transferred into the provided columns and spun at 2000 rpm for 2 minutes. Exosomal RNAs were obtained by centrifuging at 13 000 rpm for 1 minutes. The exosomal RNA integrity was evaluated by a nanodrop system (BioTek, Germany).

###  Exosomes characterization

 According to The International Society For Extracellular Vesicles (ISEV) guidelines, scanning electron microscopy (SEM) and flow cytometry analysis were used to characterize isolated exosomes. Exosomes samples (100 µL) transferred on grids and then freeze-dried for 60 min. After Au-coating, exosomes visualized by a SEM system (MIRA3 FEG-SEM, Tescan) at 30 kV. For detect exosomal marker CD63 by flow cytometry, exosomes were incubated with the primary anti-CD63 antibody for 2 hours at 4°C. Following, exosomes were mixed with secondary anti- mouse IG-FITC for 1 hour at room temperature. Finally, samples were analyzed using a BD FACSCalibur system and FlowJo software (version 7.6.1).

###  Quantitative polymerase chain reaction (q-PCR)

The expression of exosomal miRs was measured with real-time PCR (q-PCR) analysis. Firstly, exosomal RNA converted into cDNA using a commercial kit. Using a microRNA assays kit, expression of exosomal miRs was calculated. The threshold cycle (Ct) was obtained for each sample by the q-PCR system, MIC (Swiss). Expression of each miR normalized against Snord47 and the 2^−∆∆ct^ method used to measure fold changes.

###  Statistical analysis

Statistical analyses were performed using the IBM SPSS Statistics (version 16.0) software. Measurements were presented as means ± standard deviation. Comparison between two groups were analyzed by the two-sided Student’s *t* test. Statistical significance was presented as *P<0.05, **P<0.01, and ***P<0.001 versus the control group. All experiments were accomplished in triplicate.

## Results and Discussion

###  Isolated serum exosomes confirmed by SEM and flow cytometry analysis

**Figure 1:**
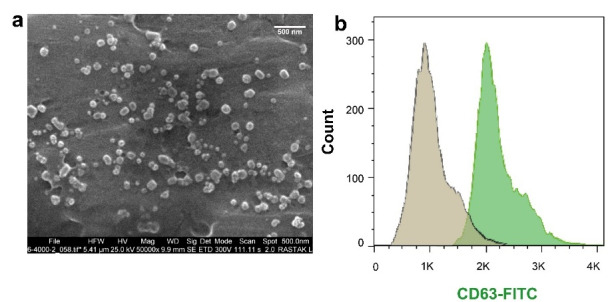
Characterization of exosomes by scanning electron microscopy (a) and flow cytometry (b)

We characterized serum-derived exosomes by SEM and flow cytometry analysis. Micrographs prepared by SEM showed nano-scale-sized and a round shape exosomes (Figure 1a). Immunophenotyping by flow cytometry confirmed CD63 marker in isolated exosomes (Figure 1b).

###  Serum exosomal miRs up-regulated in patients with breast cancer 

**Figure 2:**
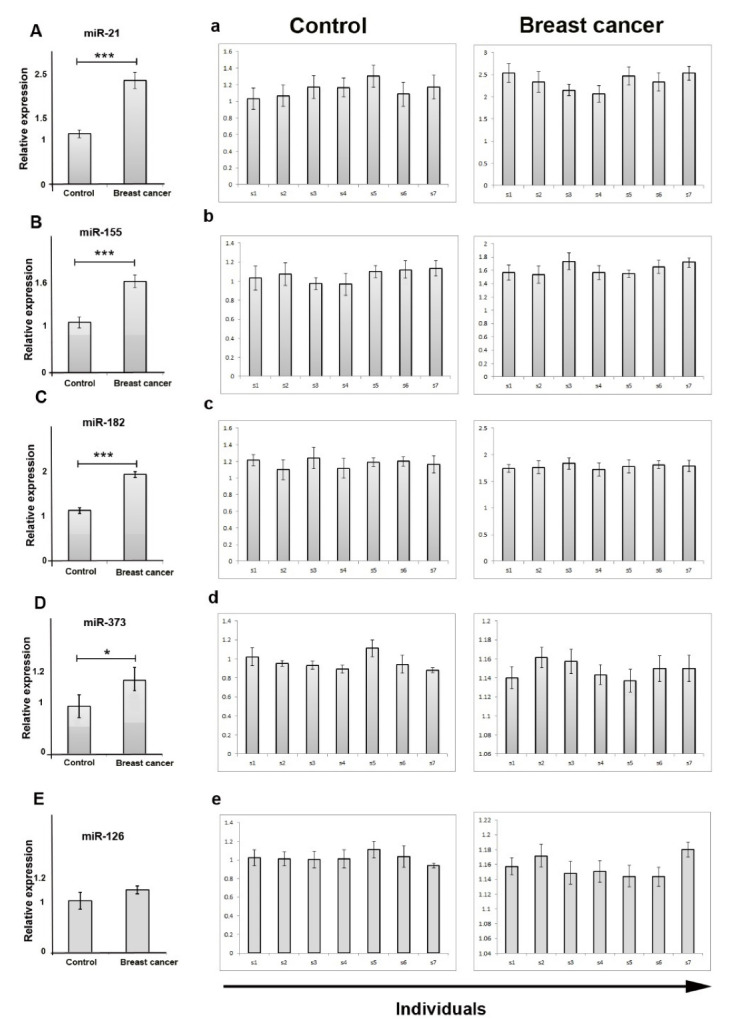
Comparison of relative expression of serum exosomal miRs between patients with breast cancer and healthy controls. Expression of miR-21 (A), miR-155 (B), miR-182 (C), miR-373 (D) up-regulated in serum derived exosomes of breast cancer patients. Right panels belong to expression of miRs in individuals (a, b, c, d, and e). Data are prepared as mean ± SD. * P > 0.05; ** P < 0.01 and ***P < 0.001. No significant comparisons are not symbolized

We compared the expression levels of the five-exosomal miRs in the serum collected from breast cancer patients and control individuals using q-PCR. We found that expression of exosomal miR-21 (*P<*0.001), miR-155 (*P<*0.001), miR-182 (*P<*0.001), and miR-373 (*P*<0.05) were considerably increased in patients (Figure 2). We also found that the relative expression levels of exosomal miR-126 were not changed significantly between groups (1.01 ± 0.09 vs. 1.13 ± 0.06; *P >*0.05) (Figure 2). Early diagnosis and treatment of cancer is the hallmark of increasing the survival rates of cancer patients.^14^ The diagnosis of breast cancer involves invasive and non-invasive approaches, which are commonly low sensitive. Exosomes have pivotal role in cell communication and regulating different bioactivities by transferring different molecules. Moreover, exosomes are thought to transfer miRs of cancer cells into the blood. Previous studies have shown that tumor cells can release exosomes into the circulation.^15^ Exosomes may more exactly react to alteration in the miRs pool in tumor cells during tumor development. Exosomal miRs may stand for biomarkers for cancer detection at an early stage.^16^ Thus, this study planned to investigate expression of serum exosomal miRs in patients with breast cancer.

In patients with breast cancer, distinct miRs are expressed unusually. For instance, expression of miR-2l, miR-10b, and miR-155 are frequently increased in tumor cells.^17^ In addition, expression of miR-30b, miR-126, miR-335, miR-17P, and miR-205 are frequently decreased in breast cancer cells.^18,19^ Abnormally expressed miRs in breast cancer have different roles; so they may act as tumor-suppressor or tumorigenesis elements. They regulate transcription factors and various signaling pathways. For example, miR-21 regulates expression of Bcl-2, an anti-apoptotic protein, as well as p53 and PDCD4; thus it is increased in serum and breast cancer tissues that correlates with increased proliferation and metastasis of breast cancer patients.^20^ However, miR-155, miR-10a, let-7e, miR-193, and miR-144 inhibit cellular apoptosis through suppressing caspase-3.^21^ miR-155 has been shown to induce the JAK/STAT3 signaling, and miR-10b down-regulates expression of E-cadherin; supporting tumorigenesis and metastasis.^22^ miR-182 may promote the development of cancer by suppressing forkhead box O1 (FOXO1).^23^

In this study, we found that all exosomal miRs unless miR-126 were significantly up-regulated in samples of patients compared to those levels in healthy controls. It was demonstrated that exosomal miR-21 is up-regulated in different cancers such as human hepatocellular carcinoma,^24^ colorectal,^25^ gastric.^26^ Exosomal miR-21 up-regulated in sera of breast cancer patients,^27^ colorectal,^28^ and esophageal.^29^ Therefore, it seems likely that miR-21 may be as a possible biomarker of breast cancer. Similarly, a high level of urine exosomal miRs such as miR-21 and miR-21-5p have been proposed as prostate and bladder cancers biomarkers, respectively.^30,31^ Consistent with He et al,we found that miR-373 and miR-182 up-regulated in serum exosomes of breast cancer patients.^32^ miR-373 is suggested to be involved in mediating various cellular processes including apoptosis, proliferation, senescence, and invasion.^33^ In breast cancer, it has been considered as metastasis-promoting miR.^34,35^ In addition, similar to our results, miR-155 was increased in blood exosomes of lung cancer^36^ and acute myeloid leukemia.^37^ In addition, expression of exosomal miR-126 was not altered in patients. Lie et al reported that expression of this miR was low in tissue samples of breast cancers as compared to healthy ones.^38^ In contrast, Volinia et al reported that expression of this miR was high in tissue samples of breast cancer cases.^39^ Grimolizzi et al suggested that miR-126 had diagnostic biomarker potential for lung cancer. They showed that expression of this miR was evaluated in serum exosomes of lung cancer patients.^40^

 Overall, given the small sample size,we think this study is preliminary investigation on the expression pattern of some miRs in exosomes of serum collected from newly diagnosed breast cancer patients. As a diagnostic tool, exosomes are stable in blood, accessible, and bioactive, thus suggesting a precise application for diagnosis of cancer. Further data collection would be needed to validate our findings.

## Conclusion

 In the present study, expression of exosomal miRs isolated from serum of patients and healthy controls measured by q-PCR, showing up-regulation of miR-21, miR-182, miR-155, and miR-373. These data may provide valuable information for the early diagnosis of breast cancer. However, we propose that further studies should be undertaken in the following areas; (i) inquiring the functional consequence of atypically expressed miRs in exosomes collected from serum of breast cancer patients; and (ii) studying biomarker potential of these miRs in a large breast cancer sample, which is one of our next major research goals.

## Acknowledgments

 Authors acknowledge the Research Vice Chancellor (VCR) at Urmia University of Medical Sciences (Urmia, Iran) for the grant.

 The Research committee at Urmia University of Medical Sciences (IR.UMSU.REC.1399.140) supported this work.

## Ethical Issues

 The ethics committee of Urmia University of Medical Sciences permitted all procedures in the present work (Ethical code: IR.UMSU.REC.1399.140).

## Conflict of Interest

 The authors declare that they have no conflict of interests.
